# Understanding the Connection Between Diet, Food Systems and Mental Health: A Qualitative Exploration of a Caribbean Small Island Developing State

**DOI:** 10.3390/nu18091427

**Published:** 2026-04-30

**Authors:** Catherine R. Brown, Cornelia Guell, Madhuvanti M. Murphy

**Affiliations:** 1 George Alleyne Chronic Disease Centre, University of the West Indies, Jemmotts Lane, St. Michael BB11156, Barbados; madhuvanti.murphy@uwi.edu; 2 European Centre for Environment and Human Health, University of Exeter Medical School, Penryn Campus, Penryn TR10 9FE, UK

**Keywords:** diet, nutrition, food security, food systems, Caribbean, SIDS, mental health, qualitative, beliefs, perceptions

## Abstract

Background/Objectives: Diet is implicated in the high burden of mental health on society, and research examining associations between these two fields is growing. However, qualitative explorations are lacking, especially within culturally diverse settings. This study aims to explore the beliefs on the mechanisms of the relationship between diet, food systems, and mental health, and the lived experience of such, through a case study of one Caribbean Small Island Developing State, to inform culturally grounded public health strategies that integrate nutritional and psychological well-being. Methods: Fifteen interviews with food system stakeholders and five focus groups with the general public were conducted. Transcripts were analyzed using a grounded theory approach with a critical realist epistemological stance. Results: Four major categories centered on beliefs of mechanistic effects of diet on mental health, as well as broader perspectives of the relationship between food systems, food experiences, and mental health. Participants believed that (1) unhealthy diets of processed and chemically treated foods contribute to poor mental health and that (2) food insecurity is a key threat to mental health, but they also believed that (3) consumption of locally produced foods and (4) residing in agricultural communities can be beneficial to mental health. Conclusions: Participants recognize that diet influences mental health through physiological, social, and structural pathways, but this connection is threatened by rising dependence on imported, processed foods. Along with complementary quantitative research, the findings highlight the potential of expanding nutritional health literacy and clinical guidance and strengthening local food systems and traditional diets for mental well-being among Caribbean Small Island Developing States.

## 1. Introduction

Tackling non-communicable diseases (NCDs) such as cardiovascular disease, cancer, and diabetes has been at the forefront of public health efforts, given its global epidemic status in most countries [1]. Though mental health has historically received less attention, it is increasingly recognized as a priority focus, holding equal importance to physical health [2,3]. The latest Global Burden of Disease estimates (2023) list mental disorders as the top-ranking cause of disability worldwide, contributing to 2123.2 years lived with disability (YLD) per 100,000 [4]. This estimate is even higher in the Caribbean, with 2530.6 YLD. Consequences of decreased economic productivity and increased costs of care—expected to cost the world economy US$6 trillion by 2030—demand that key gaps in the prevention and treatment of public mental health are understood and addressed [3].

Growing NCD incidence is linked to the nutrition transition experienced in most societies from nutrient-dense, whole foods to more processed, low-nutrient, and high-calorie foods [5]. While diet and mental health have largely been two separate fields of research, evidence on diet’s connection to mental health is growing through the field of nutritional psychiatry [6,7]. For instance, a 2022 review reports the significance of micronutrients and antioxidants in the development of some psychiatric disorders such as Parkinson’s disease, schizophrenia, and depression [8]. Anxiety and depression have been associated with systemic (including neural) inflammation from diets high in ultra-processed foods [9], while plant-based and ketogenic diets have been associated with reduced symptomatology of a range of mental health disorders, including dementia, bipolar disorder, schizoaffective disorder, and unipolar depression/anxiety [10,11,12,13,14,15].

The broader theme of food insecurity and mental health is prominent in research, with physiological hypotheses including nutritional insufficiency, psychological distress of uncertainty of food sourcing in an unsupportive food system, and shame of obtaining food in socially undesirable ways [16,17,18]. Influenced heavily by socioeconomic inequalities, food insecurity is commonly reported in low- and middle-income countries (LMICs) and Small Island Developing States (SIDS) [19,20]. LMICs pay higher tariffs on food imports than other countries, especially for fruits and vegetables [21]. SIDS are further affected by extreme weather events and economic and trade vulnerabilities, which impact food availability and accessibility [22]. The Caribbean—made up of a wide collection of SIDS and LMICs—has a 64% prevalence of food insecurity (more than double the world average) [21], which contributes to the region’s nutritional decline with poor-quality dietary intake and high burden of diet-related NCDs [23,24,25].

Despite this expanding evidence base and in support of the World Health Organization’s (WHO) claim of mental health research being “insufficient and imbalanced” [3], two major evidence gaps are apparent—location and study design. Most mental health research is conducted in high-income countries—a significant caveat given that this theme is shaped by culture, food systems, and social conditions [3,26]. Furthermore, in a review of 1895 global studies examining diet/food security and mental health, only 10 qualitative and four mixed methods study designs were found [26]. Further, this qualitative research was narrowly focused; for example, examining the lived experiences of eating with a mental disorders [27] and attitudes towards dietary changes [28,29], rather than examining beliefs on the relationship between diet and mental health and the broader food system experience, which can lay important foundations for this topic.

In the Caribbean, research examining relationships between diet and mental health is also sparse. A 2025 scoping review [30] found only four studies with qualitative components [31,32,33,34], none of which *primarily* sought to investigate this relationship. These studies offer only weak, fragmented evidence, ranging from word associations with conflict/crime and poverty/hunger [31] to modest effects of urban gardening [32], “various foods” [34], and food sharing [33] in mitigating health and climate crisis-related distress. Missing in this research is a deeper understanding of *how* people view a relationship between diet, food systems, and their mental health.

Understanding local beliefs regarding the diet–mental health nexus and the lived experience of food access and consumption is essential, as these perspectives are rooted in unique cultural contexts [3,7] and could be drivers of dietary choice and community receptivity to health interventions [35,36,37]. Many common behavior change theories, such as the socio-ecological model and the health belief model, highlight the importance of beliefs for effective behavior change [38]. In the same way that the success of dietary interventions to improve physical health can be increased by first examining and applying cultural beliefs [37,39,40], this should also be applicable to mental health. Therefore, key insights are whether and how Caribbean populations believe diet affects mental health, and their lived experience within their social and structural contexts, which may impact these beliefs.

Given the paucity of qualitative data from the Caribbean examining diet and mental health and the importance of understanding local and cultural perspectives to accompany empirical findings, this study aims to explore the beliefs on the mechanisms of the relationship between diet, food systems, and mental health and the lived experience of such, through a case study of one Caribbean SIDS, to inform culturally grounded public health strategies that integrate nutritional and psychological well-being. This study represents the first in-depth, qualitative exploration focused specifically on understanding the mechanistic beliefs about the relationship between diet, food systems, and mental health in a Caribbean SIDS.

## 2. Materials and Methods

### 2.1. Study Design

With limited qualitative research on the topic and the study’s aim of understanding beliefs and perspectives, a qualitative research design was informed by grounded theory to uncover an explanatory process about the ways people see connections between diet, food systems, and mental health [41]. The approach was guided by a critical realist epistemological stance, recognizing that participants’ accounts reflect lived experiences shaped by underlying biological, social, and food system structures [42]. The study was guided by the Consolidated Criteria for Reporting Qualitative Research (COREQ) checklist [43]. Ethical approval was granted by the University of the West Indies Cave Hill campus Research Ethics Committee (Ref. CREC-CH.00224/08/2023) and the Medical & Dental Council’s Research Ethics Committee in St. Lucia. All participants provided informed consent prior to data collection.

### 2.2. Setting

The study is nested within a large multi-setting project of the NIHR Global Health Group, Global Community Food for Human Nutrition and Planetary Health in Small Islands (GCFaH) [44]. GCFaH aims to understand the potential for improving household diet, nutrition, and food security and reducing the burden of nutrition-related diseases by promoting increased community-based food production based on agroecological principles in four small island countries.

This study was conducted in one of the participating GCFaH countries, St. Lucia. St. Lucia is a mountainous upper-middle-income SIDS in the Eastern Caribbean with a population of approximately 180,000 [45]. The ethnic background is mostly African descent (85%) and mixed (11%); English is the official language, with French/West African Creole influences spoken informally [46].

Approximately 2538 YLD per 100,000 are attributed to mental disorders in St. Lucia—above global and Caribbean estimates [4]. St. Lucia’s mental health system governance is small but expanding [47]. It has one mental health clinic, with mental health services offered in nine of the island’s 34 health centers [48]. Only 3% of its health budget is spent on mental health, all of which goes to the clinics [47]. Though mental health legislation exists—St. Lucia National Mental Health Policy (2007) and Mental Health Act (2008)—financial and human resources are yet to be allocated to allow for implementation [47,49].

St. Lucia’s land division is 19% urban versus 81% rural, with agricultural land diminishing over time [50]. The Food and Agriculture Organization (FAO) reported a 22% population prevalence of moderate to severe food insecurity in 2018 [50]. While child stunting is low (2.8% in children under five years old), nearly 7% of children are overweight and 33% of adults are obese, indicating overnutrition as an area of concern [50]. Over 80% of adults do not meet the WHO’s recommendation of 5 servings of fruits and/or vegetables per day [51]. Like many other SIDS, St. Lucia’s heavy reliance on food imports (2011 food import ratio of 83%) is believed to contribute to its population’s malnutrition [23,52].

### 2.3. Participants and Recruitment

Participants were recruited from GCFaH’s pool of existing participants who consented to being contacted by project staff for participation in other activities. Food system stakeholders were recruited for interview via email. Eligible stakeholders included adults aged >18 years who represent national, regional, and local community bodies, health practitioners and policy makers, and stakeholders from across the food value chain, such as consumers, food vendors, restaurateurs, farmers, agro-processors, supermarket managers, etc. Focus group participants (also >18 years old) were recruited by telephone. Participants were selected for recruitment with the goal of maximum variation (without oversampling) across age, gender, urban versus rural residence (focus groups), and position in the food system (interviews), since beliefs could manifest differently across these spectra.

Sampling to saturation was used with ongoing recruitment of participants during data collection and analysis. In line with grounded theory methodology, meaning saturation was assessed through constant comparative analysis using axial coding and consistent memo writing, and data collection concluded when no new concepts and linkages between diet, food system, and mental health were identified [53]. Saturation was assessed separately for interviews and focus groups as they were conducted several months apart. Ultimately, 15 food system stakeholders were recruited for interview, and 27 persons from the general public were recruited and divided into five focus groups stratified by age and gender (18–35, 36–55, 56+ years of age). The 56+ group was not gender-stratified due to challenges in recruiting sufficient numbers of persons, such as transportation barriers and ill health. Stakeholders consisted of an equal distribution of men and women who were private farmers/food processors (*n* = 6), chefs/restaurateurs/food retailers (*n* = 3), workers of the Ministries of Health or Agriculture (*n* = 4), and representatives of local/regional civil society organizations related to agriculture (*n* = 4). Including both food system stakeholders and the general public enabled the study to integrate structural, policy, and production perspectives with lived community experiences. This layering of perspectives ensured that study findings were not limited to top-down policy assessments or bottom-up anecdotal evidence, enabling data triangulation and enhancing contextual credibility of the results.

### 2.4. Data Collection and Analysis

Fifteen one-on-one stakeholder interviews were conducted online via Zoom during July–August 2024. Five focus groups (FGs) were conducted in-person at the Castries Public Library and a nearby office space in October 2024. Both followed semi-structured interview guides (see guides in File S1, Supplementary Material), and discussions lasted 45–90 min. Questions focused on the relationship between diet, food systems, mental health, and other NCDs, asking about what might contribute to mental health overall; food security, food sourcing, and mental health; and ‘food as medicine’ and connections between other NCDs and mental health. Discussions began by asking participants to describe their own definitions of mental health. However, the WHO definition [54] was read aloud to ensure a shared understanding that mental health was not just a diagnosable mental illness but that mental well-being and functional capability exist on a diverse continuum. Discussions were audio-recorded and transcribed verbatim, and field notes were made during and after each discussion. In cases where dialect is used (e.g., for herbs, food dishes), terms were translated into English after confirmation with local partners to confirm accuracy. Audio recordings were deleted immediately after transcription.

Dedoose (Version 8.0.46) qualitative software aided the analysis of transcripts using a grounded theory approach [55]. Coding followed a constant comparative method to ensure that findings remained rooted in the data, balancing theoretical sensitivity with abductive reasoning. Transcripts were initially coded (C.R.B.) using open coding, an inductive line-by-line process where codes were created without a predefined codebook. Axial coding followed by relating codes to one another. With sufficient connections made, selective (more deductive) coding was then done to develop larger categories. Memos were written throughout to track operational and theoretical ideas as the coding process moved from inductive to deductive. This process was reviewed by another researcher (M.M.M.). Finally, a member-checking triangulation approach was used by sharing the preliminary findings (in PowerPoint presentation format) with participants and asking them to reflect on whether the interpretations resonated with their experiences [56].

## 3. Results

Four major categories were developed, which centered on the mechanistic effects of diet on mental health with broader views of the relationship between food systems and mental health. They are grouped under two main headings based on their positive or negative contribution to mental health, along with diagrams summarizing perceived pathways. Of note, the topic-driven interview guide deductively instigated discussion of major concepts, such as food security, NCDs, mental distress, and local versus imported foods; however, poor-quality foods (for mental health), the personal impact of one’s own food production, and the distinct physiological mechanisms of food and of local foods emerged inductively.

### 3.1. Unhealthy Diets and Food Insecurity Are Believed to Contribute to Poor Mental Health

**Figure 1:**
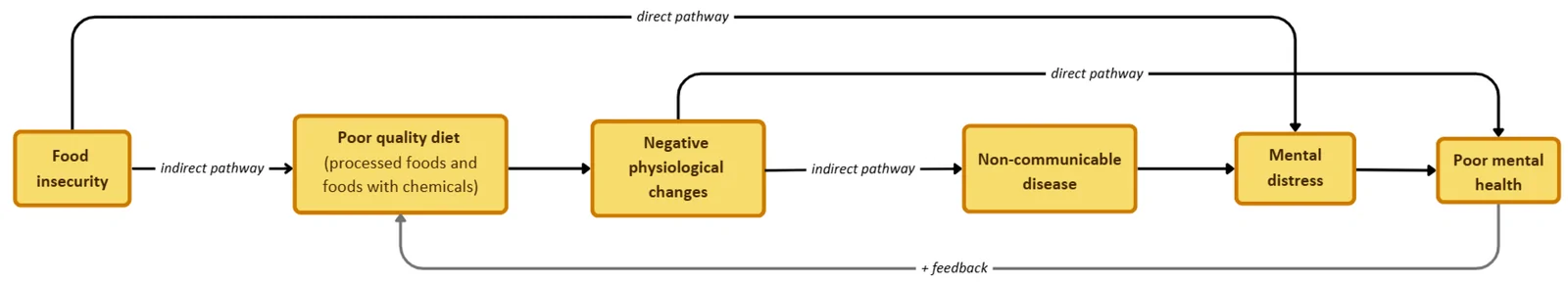
Flowchart of participants’ perceived mechanism of the effects of food insecurity and unhealthy diet on mental health.

Participants perceived that the consumption of unhealthy foods and, more broadly, food insecurity, can lead to poor mental health through direct and indirect pathways. Figure 1 illustrates these pathways, and details are discussed in the two categories below.

#### 3.1.1. Unhealthy Diets of Processed or Chemically Treated Foods Are Believed to Be Associated with Poor Mental Health

In all discussions, participants expressed assumptions that an unhealthy diet can lead to mental health symptoms such as fatigue, “*mental instability*”, brain fog, depression, and anxiety. Although severe mental health disorders were rarely mentioned, dementia was cited by one female farmer whose father is diagnosed with the condition. The unhealthy culprits were considered to be (a) processed foods, which are thought to lack nutrients and contain excess sugar and fat, and (b) foods containing agricultural and food processing chemicals, such as inorganic pesticides, fertilizers, and food additives. While participants believed strongly in the concept of ‘food as medicine’, the physiological basis of this was less conceived by most. Generally, participants held a broad belief that food’s effect on mental health was modulated by poor physical health: *“But if you don’t have a healthy body, you cannot have a healthy brain, you cannot be thinking properly.”*

(18–34-year-old male FG)

Figure 1 illustrates this perceived mechanism through two pathways: (a) direct physiological effects of a poor-quality diet leading to mental health symptomatology and (b) indirect psychological effects (mental distress) of being physically unwell due to a poor-quality diet.

The indirect pathway was the most commonly purported and implies that mental health impacts do not only come directly from food itself, but also from the experiences of being physically unwell from obesity and NCDs (which, of note, were considered by participants as physical conditions and not inclusive of mental well-being). These experiences of mental distress include the stigma of NCD diagnosis, fear of physical disability, sickness and death, financial strain of treatment, and distrust in the efficacy and safety of modern medicine (more commonly reported by older participants). The distress of being overweight due to a poor self-image and external bullying was also included, especially among youth: *“I would say diet is linked with your physical appearance because if you eat more, you gain weight and because of that, you get bullied for that. So I would say these two are linked here.”*

(18–34-year-old male FG)

*“If you get sick that’s gonna’ affect your mental health. If you get cancer, that’s gonna’ affect your mental health. If you get diabetes that’s gonna’ hurt your mental health because it’s gonna’ weigh on you on a day-to-day basis.”*

(Stakeholder 13: male beekeeper)

The direct pathway highlights the belief that unhealthy foods directly impact mental health through internal physiological processes. About one third of discussions—including two focus groups and six interviews (farmers, government officials, and one chef)—lightly referred to gut health, hormones, immune system functioning, and inflammation as mediating pathways. While one stakeholder was trained in naturopathy and spoke with confidence, others learnt about it through reading or having a family history of physical and mental health challenges: *“There are certain foods that contribute to inflammation. Okay, and mental health. Mental health can be an inflammation of the fluid in the brain inflammation of the brain. And that’s it.”*

(Stakeholder 6: female farmer with father diagnosed with dementia)

*“A long time ago, we could have churned our own butter. We don’t do that anymore…to have a better shelf-life, they add all the stuff to the food, and I think in that these are the things that change your build up, it change your hormones. You react differently…because of the hormone imbalance.”*

(36–55-year-old female FG)

A healthy diet, on the other hand, was illustrated as a buffer for day-to-day stressors, akin to a good night’s sleep. Finally, participants highlighted the bidirectionality of the relationship between diet and mental health, where poor mental health can also contribute to excess food consumption and consumption of poor-quality foods, exaggerating the cycle. Even more, once diagnosed with an NCD, participants lamented the barriers in improving physical or mental health through diet due to the limited availability and affordability of healthy foods, alongside the dominance of processed and chemically treated products on store shelves.

Lastly, participants generally did not attribute particular types of diets (e.g., vegan, ketogenic) to better mental health but rather a balanced consumption of food groups with limited processing and chemicals. However, specific foods were referenced that, together, illustrate a distinction between plant versus animal products (see Table 1). Participants most frequently identified plant-based foods—with the notable exception of yogurt—as beneficial for mental health. Conversely, while participants were less specific about foods detrimental to mental well-being, the few mentioned were predominantly animal-based products or ultra-processed items.

#### 3.1.2. Food Insecurity, Exacerbated by Reliance on Imports and Insufficient Local Food Production, Is Considered a Key Threat to Mental Health

Food insecurity was underscored as a systems-level root contributor to poor mental health in St. Lucia (see Figure 1). Participants described limited availability and accessibility of healthy foods due to insufficient local food production, high costs of healthy foods (whether imported or local), and an over-reliance on imported, processed foods—all of which were accentuated during the COVID-19 pandemic. Participants believed that food insecurity directly leads to anxiety and worry at both the household level and national level, as they considered both food items on their own kitchen shelves and on supermarket shelves.

*“But it’s just that it’s a persistent stressor. And I think that that is perhaps the most the most telling issue. It’s just a persistent stressor, and then in terms in terms of the long run, I mean. I don’t know. I don’t know how exactly [how] that might play out, but we’d expect that poor nutrition, poor health, would increase the predisposition to other health issues, including mental health issues.”*

(Stakeholder 7: male representative from regional agricultural NGO)

Further, excess money spent on costly healthy foods is also believed to impact mental health through opportunity costs of being unable to spend on other priority areas of life, such as education. One female farmer highlighted the importance of agricultural knowledge and skills in being able to manage this impact: “*Maybe you don’t know how to grow it, or you don’t have the land to grow it. So that definitely can affect our mental health.”*

Participants who work closer to the food source were particularly cognisant of what empty shelves at the supermarkets represented and the indirect effect these have: further reliance on the more available, cheaper, and lower quality, processed imported foods and subsequent poor physical and mental health.

*“And the whole issue of availability locally of course. You know, if you cannot get your food local, you are forced to go and get it outside. And even if you can’t get it, you cannot get it locally, it means that you know, it puts more pressure on more pressure on you as an individual to purchase, to purchase the imported food.”*

(Stakeholder 14: male marketer of agricultural produce)

Finally, a structural paradox was shared by a participant from the Ministry of Agriculture: if large-scale, commercial farming in St. Lucia increased, this could increase food security and subsequently help to improve mental health; however, pesticides required to consistently produce sufficient yields to meet population demands could also deteriorate mental health due to their chemical content.

### 3.2. Local Food Experiences Are Believed to Contribute to Better Mental Health

**Figure 2:**
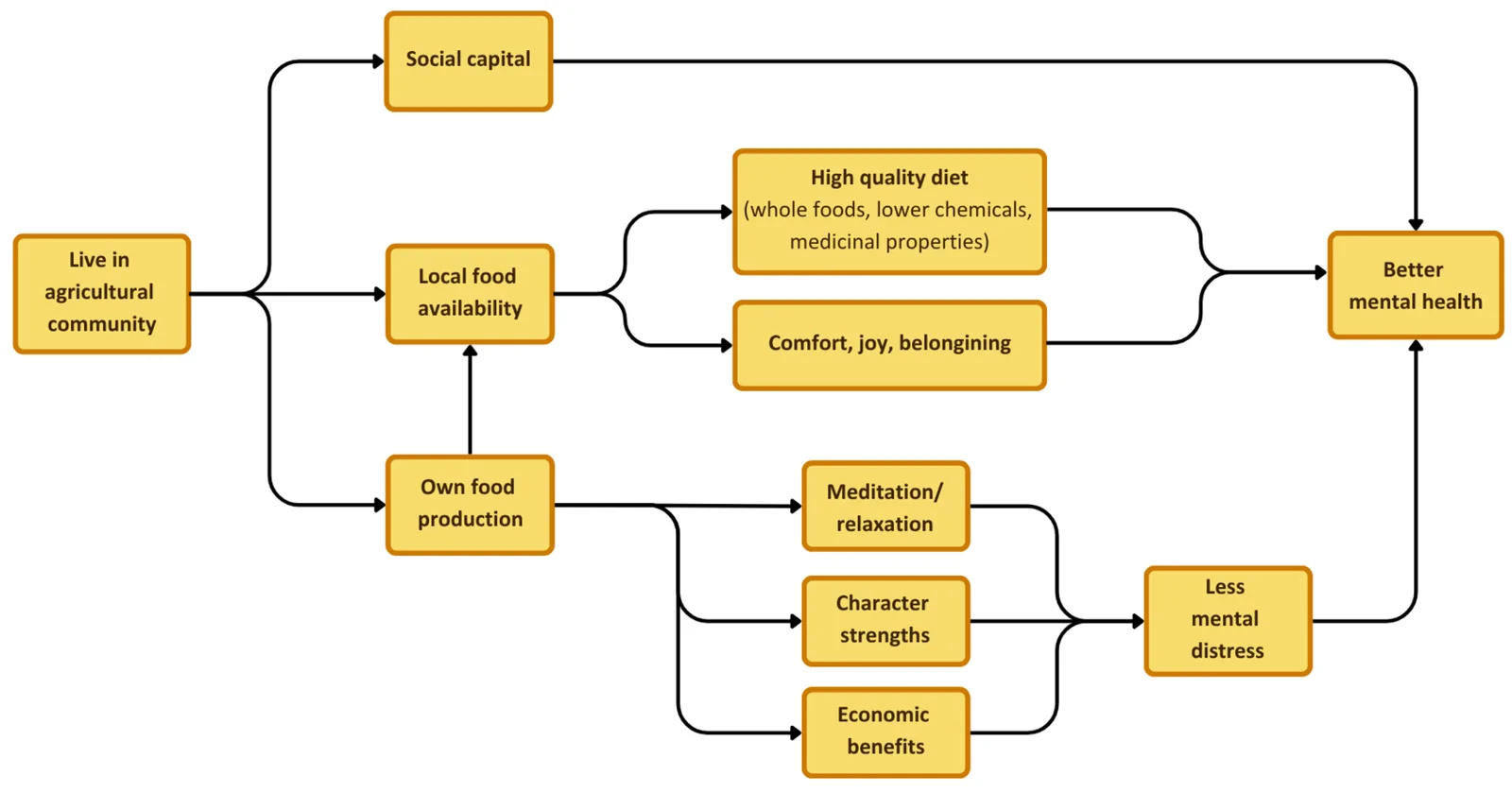
Flowchart of participants’ perceived mechanism of the effects of local food experiences on mental health.

Participants attributed better mental health to local food experiences, which include living in agricultural communities and consuming locally produced foods (regardless of residence). Figure 2 summarizes these local food experiences, which include the lived experiences of social interaction in such communities, the impact of consuming and being surrounded by local foods both at home and culturally, and the physical act of farming itself. Details are discussed in two categories, which follow.

#### 3.2.1. Rural, Agricultural Communities Are Perceived to Have Better Mental Health

Both urban- and rural-dwelling participants strongly connected food and mental health to area of residence, believing that persons living in more rural, agricultural communities have a better mental health status than urban-dwellers. Various reasons were provided, as illustrated in Figure 2. Firstly, all participants believed that these rural-dwellers have lower access to fast food outlets and better access to whole foods containing fewer agricultural and processing chemicals, and their consumption of these cleaner, locally grown foods meant healthier bodies and minds.

*“The persons in Castries is who are at the bottom of the, the economic scale would be more, more susceptible to a number of disease. And I can mention mental diseases in that right, because they do not get in the, the proper nutrition at the right time.”*

(Stakeholder 14: male marketer of agricultural produce)

Rural-dwellers were also thought to be privileged in knowing the source of their food; with less reliance on outside food retailers, participants believed this provides a sense of comfort and relief: *“With the community food, we actually know where your food come from. Because you see it growing”* (36–54-year-old male FG); *“Just the thought of knowing what you’re eating is is by itself fifty percent of your mental health already.”*

(Stakeholder 8: male farmer)

Secondly, the physical act of farming itself was also considered beneficial to mental health by reducing mental distress. Farmers in particular, and even non-farmers with childhood memories of farming with their families, described that being among greenery, in soil, and having a *“connection to earth”* was meditative and grounding to the soul, reducing stress and promoting relaxation: *“This [farming] is therapy, this is healing. This is meditation, like being in the soil, just being in the soil and growing food is meditation.”* (Stakeholder 4: female farmer) One participant enjoyed going into her garden barefoot to de-stress after a difficult day at work or fight with her partner: “*I go, I pick up tomato. I speak to the plants. I make connection with the trees. I feel good. So when I eat that, to me, it cleanse me.”*

(18–34-year-old female FG)

Yet, a range of participants noted that hardships that typically accompany farming also play a role. They believe that this can build resilience, patience, and discipline, and that these character traits spill over into other areas of life to protect one’s mental health. Further, all participants boasted satisfaction in simply reaping what you sow; being able to enjoy the fruits of your labor (quite literally) was thought to provide pride in purpose, boosting one’s mental health.

*“When you grow your own food, you feel better about eating, you get a sense of accomplishment and fulfillment, right? Because I mean, so when you see, for example, he take his watermelon and he cut it, you know he didn’t buy that—he grew that from a from a seed, right? So you feel better about it. I think it affects you mentally.”*

(36–54-year-old males FG)

The economic benefit of this reaping is another advantage, appeasing the worry and anxiety of food insecurity. All participants, but especially farmers, highlighted the money saved from not having to buy food at supermarkets thanks to sustenance farming. Finally, participants agreed that rural agricultural communities boast a strong social network and high social capital, such as neighbors to talk to, share food with, and count on in times of need.

*“So for me, you know, when you have this communal approach to the production of food, it creates a sense of community, it creates a sense of belongingness. And these are issues, or these are matters that really, I think, impact mental health and wellness as against everyone living in a high-rise building. You go to find your food somewhere in a restaurant. There’s no connectivity at that level.”*

(Stakeholder 9: male Ministry of Agriculture worker)

On the other hand, urban communities were considered more fast-paced and individualistic—characteristics thought to hinder mental health.

*“So the lack of community. It, it definitely has a negative impact on our mental health. It has a negative impact on the foods that we eat or our ability to secure foods, because again, we’ve isolated ourselves. We don’t have people around us to provide for us. We’re not in the habit of providing for others.”*

(Stakeholder 5: male representative of agricultural civil society organization)

Again, however, participants warned of the increasing trend away from local food production due to increasing urbanization.

*“We had golden apples all day long. We were never hungry. Oranges. And yeah. Ripe bananas, ripe bananas was another one. Plums. There was always a fruit that was available to us. Some were seasonal but by the time this one is finished, another one is already is being ready. So we we we had live[d] a healthy lifestyle, with foods in our environment. But today? Yes, it’s manufactured food.”*

(Stakeholder 8: male farmer)

#### 3.2.2. Local Foods (Versus Imported) Are Valued for Their Mental Health Benefits

When asked about their thoughts on locally produced foods, all participants associated their consumption with healthier physical and mental well-being than imported food. A major explanation is the belief that locally produced foods are more nutrient and antioxidant-dense, with lower concentrations of agricultural and food processing chemicals. Locally produced food was described as *“alive”* and *“medicinal”* compared to *“dead”* or *“processed”* imported foods.

*“Because when you look at the imported food, when you look at it, it have a whole level of different things in it. When we, when we grow local food, we don’t have all them labels eh?…Who knows the name of them? You find that ‘A’ that word is a poison, and other things. So it have a lot to do [with mental health].”*

(56+ both genders FG)

However, two farmers and one Ministry of Agriculture representative acknowledged agricultural chemical use in local food production and that local foods might not always be the healthiest choice. Nevertheless, participants often explained that this healthier mental well-being was also due to the simple comfort and relief of knowing where their food comes from (mentioned in Section 3.2.1).

*“But when you grow it, it’s different…and you know the fertilisers used in your crop, you know whether or not it’s natural or not. So, you know, you know what you’re eating. But whenever you go to buy it from somebody or from Massy stores, you don’t fully know what you’re eating.”*

(36–55-year-old females FG)

Participants with varied backgrounds (farmer, chef, and Ministry of Health worker) also distinguished between the transactional act of buying imported, packaged foods at supermarkets versus the more mindful, intentional joy of shopping for local, whole foods at traditional markets. Further, local foods were considered feel-good foods as participants reflected on joyful memories that eating local foods evoked—of their childhood on their grandparents’ farm or home-sourced and home-cooked meals with their parents.

*“Eating specific foods could bring you back to childhood memories, or back in the days when the family did dot dot dot [i.e., farming practices] and whole other set of emotions. So I think from that perspective, eating local traditional foods does, in fact, bring a sense of well-being. I mean, as you move from one territory to the next, you could hear the conversations around specific foods and the joy that it brings.”*

(Stakeholder 9: male Ministry of Agriculture worker)

Participants highlighted the key role local food plays in cultural identity and subsequent community unity and sense of belonging; for example, as nurtured through local food festivals.

*“It give us that sense of, yeah, we, I belong to THAT. And I think that it’s important. I mean, for our mental health. Just know that you belong. I think it’s very important not feeling like, “Hey, I’m not part of this”, or “I don’t belong here.” You know that in itself can be a stress on its own. So you know, that cultural aspect of our food…And that’s, that’s our thing. So I think it creates that sense of belonging. And I think it’s really good…It has a positive impact on our mental health.”*

(Stakeholder 15: female farmer)

While participants praised St. Lucia’s annual Creole Festival, concerns were raised about the proliferation of imported food. For example, participants interpreted KFC’s high status among youth as a loss of food culture and identity.

*“So food is an expression of cultural identity. So you know Barbados is flying fish and coo coo. Right? So you know it resonates some tradition, traditional and and traditional feelings. So food is a cultural experience….And probably we, as Caribbean people, are gonna lose our identity if we continue to function behind imported foods, like, for instance fast foods like KFC.”*

(Stakeholder 13: male beekeeper)

Participants further warned of the discouraging image some have of local food as “*poor food*”, which could lead to a higher consumption of unhealthy imported foods (such as chemical-filled fruits or KFC); although, on the other hand, their higher costs and accompanying image of higher economic standing could *“boost your self-esteem…and your mental health”* (18–34-year-old male FG). However, this same focus group believed this to be overshadowed by the growing concern of chemicals and the low nutrient quality of these same imported foods. Yet, one Ministry of Agriculture stakeholder argued that the discussion of food sourcing and mental health is irrelevant due to his belief in the island’s unavoidable need to import to keep up with national food demands.

Finally, generational food preferences emerged during discussions on local versus imported foods. Despite all age groups appreciating the value of locally produced foods for mental well-being, younger adults tended to prefer imported and processed foods, such as KFC or ice cream, as their immediate mood-enhancing comfort foods. Older adults preferred more local whole foods, such as coconut, fruits, teas, and whole food dishes, like green fig and saltfish. Additionally, older adults more commonly touted the medicinal properties of local foods and organic farming techniques than younger adults. In fact, showcasing of local foods through food festivals and creative chefs were considered vital in maintaining younger generations’ interest in traditional foods:

*“Instead of just cooking green figs, green bananas just like that, they would make banana salad or make different things with the bananas…as opposed to just cooking…or roasting the breadfruit…which a lot of the younger generation they do not buy.”*

(Stakeholder 10: male food processor, previously Ministry of Agriculture)

## 4. Discussion

### 4.1. Summary of Findings

This study qualitatively explored beliefs on the connection between diet, food systems, and mental health from food system stakeholders and the general public in a Caribbean SIDS, St. Lucia. Insights from both groups of participants converged into four central categories of beliefs on diet and food systems’ effects on mental health. Mental health is believed to be negatively impacted by a poor-quality diet via physical changes in the body, directly through physiological mechanisms and indirectly through the mental distress of living with NCDs; food insecurity is a major threat to mental health through similar direct and indirect pathways. Also, local food experiences are believed to benefit mental health. Residing in agricultural communities is thought to offer improved social capital and direct personal benefits of one’s own food production. Participants perceived strong mental health benefits of consuming higher quality local food, and the experiences of such foods at a personal and cultural level.

### 4.2. Findings in Context

This is the first published in-depth exploration of the beliefs on mechanisms and lived experiences of the relationships between diet, food systems, and mental health in a Caribbean SIDS. It expands the location, study design, and focus of limited diet and mental health research, both regionally and globally. It represents an endeavor prioritized at the highest government level by SIDS heads of state across the region, who have recognized the threat of NCDs and mental health on development and the need for context-specificity and traditional knowledge in addressing underlying drivers of risk [57].

While the concept of ‘food as medicine’ resonated with participants holistically, the underlying physiological mechanisms were less understood by most. Existing qualitative research on this topic limits the comparison of these beliefs to other settings. For instance, limited evidence indicates that foods have been seen as “fuel for the brain” or as emotional or behavioral triggers, but this was mostly through the lens of parents assessing children’s behaviour [27,58,59]. Also, broader qualitative studies examining healthy eating in general show preliminary evidence of beliefs of the intersection of diet and holistic well-being [60]. However, quantitative biological evidence purports several mechanisms of action of diet’s impact on physical and mental health. These include nutrient deficiency, oxidative stress and inflammation, and the gut microbiome and gut–brain axis [9,61,62]—examples of which were mentioned by a minority of participants. For example, gut dysbiosis is associated with increased susceptibility to a variety of conditions, including traditional physical NCDs, such as diabetes and cancers, as well as mental health conditions, including anxiety, depression, bipolar disorder, Alzheimer’s disease, and autism spectrum disorders [62,63,64,65]. The syndemic framework has been used to explain the simultaneous epidemics of physical and mental health disorders, which share key determinants and are themselves risk factors for one another [66].

Therefore, conceptualizing beliefs about the mechanisms of action of food on health also requires a deeper analysis of the food system, specifically how systemic structures act as intervening mechanisms. For instance, it is estimated that 80–90% of food consumed in the Caribbean is imported, with only three countries (Guyana, Belize, Haiti) producing more than 50% of their own food [67]. Furthermore, the cost of a healthy diet in the Caribbean is 42%, 13%, and 5% higher than the averages for the Organization for Economic Co-operation and Development countries, LMICs, and other SIDS, respectively; furthermore, more than half of Caribbean populations are unable to afford these costs [67,68]. It is unsurprising, then, that food insecurity and local food, such as within agricultural communities, feature prominently in pathways perceived to affect mental health (Figure 1 and Figure 2). Mental distress stemming from unreliable food supplies and an over-reliance on imported foods aligns with global evidence linking food insecurity to psychological distress, depression, and anxiety [16]. However, food security is defined not only by food availability and accessibility but also by food preferences [20], highlighting the significance of local food preferences found in this study. And though food security refers to nutritious food by definition, it is worth considering whether the growing availability of and reliance on less nutritious convenience foods might eventually create an illusion of food security for laypersons. That is, if a fruit or vegetable is unavailable, for example, convenience food may easily take its place on market shelves, possibly alleviating worry and anxiety regardless of its health impact.

The value of local foods over imported alternatives (in general) has been reported in other SIDS, with similar attributing themes of the nature of the food itself and wider cultural practices, including pesticide content and cultural pride [69]. This study builds on this previous research by specifically identifying perceptions of mental health benefits. Consistent with studies on the protective effects of traditional diets [10], older participants in this study demonstrated a nuanced understanding of the link between nutrient-dense, plant-based foods and mental well-being (Table 1) [10]. However, whether locally produced foods are in fact healthier due to lower chemical content than food imported into St. Lucia is unknown. Nevertheless, the characterization of local foods as *“alive”* and *“medicinal”* reflects a deeply rooted connection to food heritage and identity, which has been shown to promote mental well-being [70,71,72]. Food is considered a marker for identity and social cohesion, so the transition from traditional to imported foods represents both a nutritional shift and a disruption of the “self” that may act as a generative mechanism for psychological distress [73,74,75]. Yet younger participants’ preference for processed foods as comfort foods in spite of their beliefs of its mental detriments points to the deepening reach of food industrialization and weakening belief–behavior relationships in changing food environments [76]. These generational differences are substantiated by quantitative evidence: greater than three times more younger age groups consume more high-salt processed foods than older age groups in St. Lucia [51].

The perceived mental health advantages of rural, agricultural lifestyles underscore broader social determinants of mental health, as they relate to land and food access, food safety, and social capital. Specifically, farmers’ reported connection to nature resonates with evidence supporting mental health benefits of green spaces and outdoor activities [77]. Mindfulness/relaxation experienced by farmers during food production, as well as mindful shopping at traditional local farmers’ markets, align with principles of mindfulness that are increasingly recognized as beneficial for mental health [78]. Further, agricultural wealth has been shown to better predict mental well-being than the cash economy in rural areas of Haiti, and success in such might further enhance social capital, further buffering mental well-being [79]. The perceived pride and resilience derived from sustenance farming highlight the psychological value of self-sufficiency and sustainability, supporting similar findings in another Caribbean SIDS [72]. Nevertheless, international primary research has found contradictory associations between occupational farming and mental health—citing financial hardship, work demands, climate change, pesticide exposure, and loneliness as contributing factors; however, secondary reviews of comparative studies (farmers versus other occupations) report mixed results [80,81,82,83]. Context is likely a major contributor to differing outcomes. Caribbean SIDS, for example, have climatic vulnerabilities, smaller farms (e.g., more backyard gardens), and a unique cultural lifestyle within which they work and live as farmers and accommodate hardships [72,80]. Participants’ beliefs about farming are perhaps tempered by the local context of smaller-scale farming in close-knit communities. And notably, only some participants of this study were current farmers themselves who could speak from direct experience.

Finally, the way participants understand the links between diet, food systems, and mental health reflects small models of perception (Figure 1 and Figure 2). While not necessarily representing a fully developed grounded theory model, they reflect layers of the socio-ecological model of health [84]. Intrapersonal examples of how people see diet/food system impacting mental health include, for example, beliefs related to food content itself and its biological pathways; personal effects of own food production; and the direct joy and comfort imparted by local foods. However, these intrapersonal factors are inherently shaped by perceived outside layers, including factors at the interpersonal (e.g., social capital in agricultural communities), institutional (e.g., local food availability), and community level (e.g., community belonging and unity from local food experiences). All of these are ultimately governed by the furthest policy/system layer—food security. Food security is itself an outcome of wider structural elements, such as trade regulations or agricultural policies, that dictate which foods are available and accessible. This perceived interconnectedness demonstrates that empirical relationships between diet and mental health are not merely products of personal choice in consumption but also outcomes of a multi-dimensional system of balancing and/or reinforcing elements across levels of the socio-ecological model of health. Subsequently, while this framework could reveal multiple entry points for possible intervention—ranging from psychological support to systemic policy reform—it also highlights the difficulty of achieving lasting change, as improvement at one level can be undermined by failure in another.

### 4.3. Implications

The study does not try to claim that participants’ understandings should be read as definitive, empirical evidence. Rather, exploring beliefs can inform communication and education around healthy foods, and understanding lived experiences can help highlight social determinants and nuances in how such mechanisms are experienced.

At the highest level, the concern of food availability underscores the need to strengthen St. Lucia’s local food system to reduce the perceived mental health toll of food insecurity, especially given increasing urbanization, trade liberalization, and potential influx of convenience (processed) food in the island [85,86]. It is recommended that food system interventions in SIDS focus on an enabling environment politically and institutionally; sustainable and resilient management practices; and community empowerment [72,87]. While not originally designed specifically for health promotion, CARICOM’s regional initiative to reduce food imports by 25% by 2030 remains a pillar of hope for encouraging the production and consumption of locally produced food and, possibly, better perceived mental health [88]. Community-level initiatives to enhance food security could simultaneously strengthen local food production while addressing both nutritional inadequacies and perceptions of their effects on mental health [23]. For example, the FOA’s recent Resilient Caribbean Initiative linking St. Lucia’s National School Feeding Program with local food producers has helped boost capacity and skills among farmers through training while improving thousands of children’s nutritional intake; though budgetary constraints and infrastructure remain challenges over time [89]. Additionally, the regional Caribbean Agricultural Research and Development Institute and St. Lucia’s Ministry of Agriculture are working together to provide support for establishing home food systems as part of broader food security strategies [90]. Caribbean research indicates that perceived barriers to engaging in home gardening (e.g., lack of experience, expenses, weather unpredictability) are surmountable relative to perceived benefits (e.g., financial gains, food safety, social well-being) [69,91,92,93]. Importantly, however, such policy-wide initiatives must be culturally tailored, not only to enhance cultural acceptability but also to strengthen climate resilience; for example, by prioritizing indigenous foods and sustainable agricultural practices (water harvesting, greenhousing/hydroponics, regenerative farming), ensuring that locally grounded impacts on mental health are recognized [30,70,72,94].

However, a robust system alone is not enough. Even with the right policy agenda, powerful transnational companies continue to encroach on local food practices in SIDS, within a backdrop of changing generational food preferences and national declines in agriculture [50,72]. To counter this, systemic efforts must be complemented with deeper insights at individual and community levels. There is a significant opportunity to capitalize on community beliefs to tailor public health interventions. As suggested by participants, celebrating local food festivals and using more creativity in local food preparation might help maintain youth interest in local foods and cultural identity. Furthermore, the dominating narrative that psychological distress often arises from physical illness (Figure 1) suggests that interventions addressing chronic disease management and psychosocial support could help to mitigate perceived diet-related mental health burdens. Given that some participants accurately referenced elements of physiological mechanisms, improving public health literacy on the physiological connection between diet and the brain (within the whole body system) could be studied to see whether it improves receptivity to dietary changes as part of overall health promotion and as part of mental health care specifically [95]. For example, primary and secondary school curricula on nutrition could be expanded to also include mental health impacts of food, and mental healthcare provider training could incorporate modules on nutritional psychiatry. Healthcare providers could engage more effectively with patients by framing dietary changes as an adjunct to conventional treatment while correcting misinformation (e.g., superfood myths).

Finally, while international quantitative evidence exists to support participants’ mechanistic beliefs that diet and food security can impact mental health, further research could verify the perceived mental health benefits of consuming locally produced foods (including their chemical and nutrient content) and residing in agricultural communities. Complementary qualitative work could examine the knowledge, attitudes, and practices of mental health practitioners to see whether they align with stakeholders and the general public of this study and/or with tenets of nutritional psychiatry. Belief–behavior studies could also show the extent to which holding the belief that diet affects mental health actually translates to better dietary choices. Understanding this belief–behavior gap could further highlight structural barriers (such as food security, as found in this study) that prevent healthy eating despite beliefs about the mental health impacts of healthy foods. Finally, this study demonstrates that any experimental studies examining the impact of interventions promoting local food production should include mental health indicators among their outcomes.

### 4.4. Study Strengths, Limitations, and Reflexivity

A common thread in mental health advocacy is improving the mental health evidence base [96]. In addition to its wider contribution to better understanding local contexts surrounding diet, food systems, and mental health in Caribbean SIDS, this paper also contributes to a small pool of peer-reviewed published research on mental health in St. Lucia [48,97,98,99]. The study sample was also well-rounded by key demographic factors—age, gender, residence, and background in the food system (high-level policy makers to front-line retailers and consumers)—which strengthens the relevance of the findings for culturally appropriate and actionable interventions. Using a critical realist stance—operationalized through this well-rounded sample and an interview guide asking about simple beliefs and deeper mechanisms—allowed this analysis to move beyond a simple understanding of beliefs about mechanisms of food on mental health but also deep-seated social, cultural, and economic mechanisms that might generate such beliefs. Several forms of triangulation (several researchers, triangulation of stakeholder and general public findings, and member-checking of findings) also strengthen the rigor and trustworthiness of interpretations. Further, the convergence of stakeholder and public perspectives indicates a shared socio-cultural understanding of relationships, which likely reflects a collective exposure to similar food–environment pressures and supports the implementation of integrated, community interventions.

Limitations (or considerations) of the study lie in the study sample composition, mental health stigma, and researcher analysis/reflexivity. Any study on mental health is at risk of Neyman bias, where persons with poor mental health may disproportionately refuse to participate in (or withdraw from) the study. These persons with lived experiences might offer an angle to the topic not captured here. The stigma of mental health itself might have limited the depth of discussions (especially in focus groups), keeping the discussion on mental health as an abstract whole rather than specifically on mental health disorders. Although recruitment from an ongoing nutrition and food security study may over-represent individuals with a pre-existing interest in such themes, this approach was still important to secure a purposive sample of information-rich participants whose pre-existing engagement with food systems allowed for a more nuanced exploration of complex dietary mechanisms. However, acknowledging the roles of researcher reflexivity and theoretical sensitivity [100], the researchers emphasize that their academic backgrounds in nutrition and food security and demographic differences from St. Lucian participants shaped the production of knowledge within a critical realist framework. While researchers’ expertise in food security helped identify key categories, it also carries the risk of foregrounding familiar explanatory frameworks [42]. Another limitation is investigator triangulation—although the analysis process was reviewed in real-time by another researcher, double independent coding by multiple researchers could have strengthened the credibility of the findings further. Ultimately, the resulting theories are presented as a contextualized, partial explanation of local food systems and mental well-being that remains open to further empirical development.

## 5. Conclusions

This study demonstrates that St. Lucians widely perceive diet and food systems as important determinants of mental health, operating through physiological, social, and structural pathways. Food security and local food experiences are believed to facilitate food’s potential positive impact on mental health, but generational shifts toward processed foods and increasing regional dependence on imports are believed to threaten these perceived protective relationships, particularly in the context of structural vulnerabilities faced by SIDS. These perceptions have important implications for ensuring culturally grounded policy, practice, and future research. Strategies should incorporate these beliefs, such as continuing research to expand and validate beliefs, improving public health literacy on diet’s impact on mental health, tailoring clinical guidance for patients, and strengthening local food systems.

## Figures and Tables

**Table 1:** Examples of foods believed by participants to affect mental health.

Food Category	Foods Good for Mental Health	Foods Bad for Mental Health
Fruits and vegetables	* Fruits and vegetables, as a food group. * Cucumber (calming). * Coconut-oil, milk, or dried (memory, mental stability).	
Herbs	Mint (relaxation). *Bacopa monneiri* (memory, attention/hyperactivity). Rosemary (memory). Stinging nettle (memory, relaxation). Holy basil/tulsi (memory, relaxation). Broad leaf thyme. Chamomile (relaxing).	
Other plant leaves	Sour soup leaf (memory, relaxation). Guava leaf. Green tea (relaxing).	
Animal products	Yoghurt.	Cow milk. Animal bone.
Nuts and seeds	Walnuts (brain health).	
Seasoning/spice	Turmeric.	
Micro-/macro-nutrients and Other	Omega-3 fatty acids. Kombucha.	* Processed food with excessive salt (on edge, quick to upset, aggressive). * Processed food with excessive sugar (*tired, unstable mentally, hyperactive, cannot concentrate, anxious, dementia*).

Footnotes: Specific effects, where mentioned, are listed in brackets; *—commonly quoted foods (in 5 or more transcripts).

## Data Availability

The qualitative data generated and analyzed during the current study are not publicly available due to the small size of the study population and the risk of compromising participant anonymity. Inquiries regarding the data can be directed to the corresponding author.

## Supplementary Materials

The following supporting information can be downloaded at: https://www.mdpi.com/article/10.3390/nu18091427/s1, File S1: Interview Guide for Stakeholder Interviews.

## Abbreviations

The following abbreviations are used in this manuscript:

FAO	Food and Agriculture Organization of the United Nations
LMIC	Low- and middle-income country
NCD	Non-communicable disease
SIDS	Small Island Developing State
WHO	World Health Organization
YLD	Years lived with disability
