# Supplementary material for: Understanding the Connection Between Diet, Food Systems and Mental Health: A Qualitative Exploration of a Caribbean Small Island Developing State
Source: Nutrients. 2026 Apr 30;18(9):1427. doi: 10.3390/nu18091427 (PMC13164642; doi:10.3390/nu18091427)
Supplement: Supplementary file 1 [file nutrients-18-01427-s001.zip › nutrients-4245784-supplementary.pdf]

<u>Interview Guide for Stakeholder Interviews</u>

Focus: Nutrition / Food / Agriculture / Food Systems & Mental Health

1. Tell me a bit about **your work** and how it relates to the food system?

2. When I say **"mental health",** what does that mean to you?
    a. At the end of this discussion: define mental health as per WHO: *"Mental health is a state of mental well-being that enables people to cope with the stresses of life, realize their abilities, learn well and work well, and contribute to their community. Mental health is more than the absence of mental disorders. It exists on a complex continuum, which is experienced differently from one person to the next, with varying degrees of difficulty and distress and potentially very different social and clinical outcomes." (Taken from Mental Health, WHO 2022, https://www.who.int/news-room/fact-sheets/detail/mental-health-strengthening-our-response)*

3. Thinking about all aspects of health - for example, physical, mental, emotional, spiritual, social - so thinking about all of these, how do you think the **food system impacts our health**?

4. How do you think **food security** impacts our mental health, if at all?
    a. Availability, accessibility, utilisation, stability, agency and sustainability?
    b. Are there vulnerable groups? Protective factors? Risk factors?
    c. Examples from your work?

5. Do you think **local food (vs imported food)** plays a role in our mental health? In what way?
    a. Types of foods?
    b. Are there local/traditional foods that evoke certain feelings?
    c. Examples from your work?

6. Food can be sourced in different ways - purchased, grown on your own, borrowed or shared from others. Do you think how you **source your food** is connected to your mental health and well-being?
    a. How does growing our own food affect our health? Physical? Mental? Emotional? Spiritual? Social?
    b. Examples from your work?

7. How do you think **communities** affect how we interact with food?
    a. Do you think it is different urban VS farming communities? Do you think mental health differs between these types of communities?
    b. Sense of community?
    c. Examples from your work

8. Have you heard of the phrase **"food is medicine"**? How do you interpret this? Do you think it also can apply to mental health?
    a. Any types of diets in particular are best?
    b. Any mental health conditions?
    c. Examples from your work
    d. In addition to WHAT we eat, is there a role of HOW we eat?

9. How do you think **culture** plays a role in how we think about the relationship between diet and mental health?
    a. Is there something specific to the St. Lucia culture?
    b. Examples from your work?

10. So we have seen that **mental health plays a role** [or does not play a role] in the food system. But how can we integrate it more?
    a. Where do you think SLU fits in this?
    b. Types of foods? Food security? Food sourcing? Food growing?
    c. Examples from your work?

<u>Interview Guide for Focus Groups</u>

Focus: Food / Mental health / Physical health

1. When I say **"mental health",** what does that mean to you?
    a. At the end of this discussion: define mental health as per WHO: *"Mental health is a state of mental well-being that enables people to cope with the stresses of life, realize their abilities, learn well and work well, and contribute to their community. Mental health is more than the absence of mental disorders. It exists on a complex continuum, which is experienced differently from one person to the next, with varying degrees of difficulty and distress and potentially very different social and clinical outcomes." (Taken from Mental Health, WHO 2022, https://www.who.int/news-room/fact-sheets/detail/mental-health-strengthening-our-response)*
    b. How does this fit in with your interpretation of mental health we discussed earlier?

2. What are some things that you think **affect our mental health**?

3. Do you think that your **diet is related to your mental health**? How so?
    a. (If no) In what way might your diet relate to your mental health?
    b. Can you give me one example, or one memory, of when food was impacting on your mental health?

4. Do some foods **make you feel happy**, or any other positive state?
    a. Thinking about timeframe, is there a difference short-term (immediately when you eat it) vs long-term (an hour or weeks later)?

5. Do some foods **make you feel unhappy**, or any other negative state?
    a. Thinking about timeframe, is there a difference short-term (immediately when you eat it) vs long-term (an hour or weeks later)?

6. Food can be sourced in different ways - purchased, grown on your own, borrowed or shared from others. Do you think how you **source your food** is connected to your health and well-being?
    a. How might growing our own food affect our health? Physical? Mental? Emotional? Spiritual? Social?
    b. How might sharing our food affect our health? Physical? Mental? Emotional? Spiritual? Social?
    c. Sense of community?

7. Do you think there is a relationship between **physical health and mental health**? How would you describe it?

8. Do you think there is a relationship between **mental health and NCDs** like diabetes, cardiovascular disease, camera or obesity?
    a. What about risk factors like….?
    b. What do you think the basis of this is?

9. Have you heard of the phrase **"food is medicine"?** How do you interpret this? Do you think it also can apply to mental health?

10. How do you think **culture plays a role** in how we think about the relationship between diet and mental health?
    a. Is there something specific to the St. Lucia culture?
    b. Do you think local food (vs imported food) plays a role in our mental health? Are there local/traditional foods that evoke certain feelings?
